# Self-actuated biomimetic nanocomposites for photothermal therapy and PD-L1 immunosuppression

**DOI:** 10.3389/fchem.2023.1167586

**Published:** 2023-03-17

**Authors:** Wenxin Li, Fen Li, Tao Li, Wenyue Zhang, Binglin Li, Kunrui Liu, Xiaoli Lun, Yingshu Guo

**Affiliations:** ^1^ Shandong Provincial Key Laboratory of Molecular Engineering, School of Chemistry and Chemical Engineering, Qilu University of Technology (Shandong Academy of Sciences), Jinan, China; ^2^ School of Chemistry and Chemical Engineering, Linyi University, Linyi, China

**Keywords:** biomimetic nanocomposites, platelet membrane, horseradish peroxidase, photothermal therapy, immunotherapy

## Abstract

Biomimetic nanocomposites are widely used in the biomedical field because they can effectively solve the problems existing in the current cancer treatment by realizing multi-mode collaborative treatment. In this study, we designed and synthesized a multifunctional therapeutic platform (PB/PM/HRP/Apt) with unique working mechanism and good tumor treatment effect. Prussian blue nanoparticles (PBs) with good photothermal conversion efficiency were used as nuclei and coated with platelet membrane (PM). The ability of platelets (PLTs) to specifically target cancer cells and inflammatory sites can effectively enhance PB accumulation at tumor sites. The surface of the synthesized nanocomposites was modified with horseradish peroxidase (HRP) to enhance the deep penetration of the nanocomposites in cancer cells. In addition, PD-L1 aptamer and 4T1 cell aptamer AS1411 were modified on the nanocomposite to achieve immunotherapy and enhance targeting. The particle size, UV absorption spectrum and Zeta potential of the biomimetic nanocomposite were determined by transmission electron microscope (TEM), Ultraviolet-visible (UV-Vis) spectrophotometer and nano-particle size meter, and the successful preparation was proved. In addition, the biomimetic nanocomposites were proved to have good photothermal properties by infrared thermography. The cytotoxicity test showed that it had a good killing ability of cancer cells. Finally, thermal imaging, tumor volume detection, immune factor detection and Haematoxilin-Eosin (HE) staining of mice showed that the biomimetic nanocomposites had good anti-tumor effect and could trigger immune response in vivo. Therefore, this biomimetic nanoplatform as a promising therapeutic strategy provides new inspiration for the current diagnosis and treatment of cancer.

## 1 Introduction

So far, cancer remains a big problem in the field of medicine. Although scientists continue to explore and have made great achievements in this field, there are still many unknown questions to be solved. Overcoming drug resistance and recurrence is a major challenge in overcoming cancer (Boehnke et al., 2022). At present, the commonly used treatment methods include surgery, chemotherapy, phototherapy, immunotherapy and so on. However, due to the particularity of tumor microenvironment (TME), the effects of various tumor treatments are limited. Exploratory studies have determined that single therapy is difficult to achieve good tumor elimination. Therefore, in order to improve the antitumor efficacy and tumor recurrence and metastasis, multimodal collaborative therapy is urgently needed.

Photothermal therapy (PTT), as a new method of disease treatment, has attracted extensive attention because of its unique advantages. PTT is a new and less invasive method for tumor treatment, which uses the ability of photothermal agent (PTA) to convert light energy into heat energy to kill tumor cells under external light sources such as near-infrared (NIR). Compared with other traditional tumor treatment methods, PTT has obvious advantages such as no ionization toxicity, no drug resistance, and low toxic side effects. Therefore, many PTAs have been explored as options for enhancing photothermal conversion efficiency to achieve better therapeutic outcomes. Currently available PTAs mainly focus on gold, silver, palladium based nanomaterials, carbon based graphene, carbon tubes, transition metal sulfide and oxide and other inorganic nanomaterials. However, there are some unavoidable problems in the application of these nanomaterials in photothermal therapy, such as poor biological metabolism, toxicity, complicated synthesis steps and high cost. Prussian blue nanoparticles (PBs), as an ancient blue dye, have a specific photothermal effect due to charge transfer within the crystal, which can effectively convert red and NIR light into heat, and can be used for PTT in cancer treatment (Xue et al., 2018; Yang et al., 2019). PB can be used as a stable and strong photothermal reagent, which overcomes the shortcomings of many traditional nanomaterials, such as complex preparation, high price and low biosafety, and is widely used in the biomedical field. PB, as an inorganic material, does not have the characteristics of targeted diagnosis and treatment sites. Therefore, the PB with good photothermal properties can be further modified with biomolecules (including biofilms, proteins, nucleic acids, etc.) to achieve effective diagnosis and treatment of cancer. This multifunctional Prussian blue nanocomplex will have a profound impact on the biomedical field. For example, Li et al. (2020a) prepared PB/polyacrylic acid/gold aggregated nanoparticles for enhanced PTT, chemotherapy, and CT imaging, which has attracted increasing attention. It is worth noting that PB usually has no drug loading space due to its smooth and dense surface. In the process of cancer treatment to overcome the shortcomings of PBs are not easy to modify, which is the key to improve the therapeutic effect (Zhang et al., 2019a; Yu et al., 2021).

In order to achieve the effective delivery and long circulation characteristics of nanomaterials *in vivo*, inspired by natural cells, the materials formed by wrapping nanomaterials in the inner core using cell membranes as carriers are called endogenous membrane biomimetic nanocarriers (EMBNs) (Guo et al., 2022). The technology is widely used in drug delivery and cancer treatment (Guo et al., 2021a; Guo et al., 2021b; Guo et al., 2021c; Qu et al., 2021; Srivastava et al., 2022). Because these EMBN retained the original protein, antigens and immune part of the cell membrane, so in addition to high biocompatibility, also have different characteristics. Red cell membrane (RBCM) can enhance the cycle time of biomimetic nanocarriers, cancer cell membrane (CCM) can actively target biomimetic nanocarriers to homologous cancer cells, and platelet membrane (PM) can enable biomimetic nanocarriers to have immune escape ability (Guo et al., 2020; Chen et al., 2021). It is worth noting that platelets (PLTs) play an important role in tumorigenesis and metastasis. It is clear that PLTs are not merely bystanders of the circulatory system, but functional participants in all steps of primary tumor growth and metastasis. As circulating sentinels in the blood, PLTs also respond to vascular damage and invading microbes. Recent advances in biomaterials have shown that EMBN produced by PMs have some significant advantages, such as reduced macrophage uptake and enhanced immune escape ability, as well as specific targeting to cancerous and injury sites (Zhang et al., 2020a; Wang et al., 2020; Kong et al., 2022).

Recently, some scholars have found that high temperature can promote immunogenic cell death (ICD) of tumor cells and produce tumor-associated antigens to stimulate immune response in the process of hyperthermia induced PTT therapy (Xie et al., 2019; Wang et al., 2022). The specific mechanism of immune response during hyperthermia induced PTT treatment is that necrotic or programmed death tumor cells stimulate immature dendritic cells to transform into mature dendritic cells, and then mature dendritic cells present antigen to cytotoxic T cells (CTLs). When activated, CTLs can undergo a second round of cancer cell clearance. In addition, the emergence of immune checkpoint inhibitors provides a new idea for improving the anticancer effect of synergistic therapy. At present, tumor immunotherapy mainly refers to programmed death 1 (PD-1) and programmed cell death-Ligand 1 (PD-L1) antibodies and chimeric antigen receptor T-cell immunotherapy (CAR-T) for the treatment of refractory or relapsed acute B lymphoblastic leukemia. PD-1 is found primarily on the surface of activated T lymphocytes. PD-L1 is a ligand of PD-1, and its function under normal circumstances is to bind to PD-1 on the surface of T cells, acting as a “brake” to prevent T cell activation. “Cunning” tumor cells have evolved to selectively overexpress PD-L1 to suppress T cell function and allow them to escape immune surveillance (Zhou et al., 2021). Blocking the binding of tumor cells to T cells and freeing T cells is the main effect of PD-1/PD-L1 inhibitors. When the immune system’s offensive power is restored, T cells can take up arms, reidentify tumor cells and attack and kill (Liu et al., 2017; Yan et al., 2019; Zhang et al., 2020b). However, in the process of antibody development, there are strict technical requirements, and technical bottlenecks limit the production and function of antibodies. The development of antibodies not only consumes a lot of costs, but also the repeatability of the same batch and the long-term stability of antibodies are difficult to be guaranteed. Aptamer as powerful binding molecules, have some unique advantages in addition to the affinity and specificity of monoclonal antibodies. First of all, aptamer has high affinity, strong specificity, easy to prepare, and has the advantages of low immunogenicity and toxicity, stable chemical structure, not easy to be affected by environmental factors and easy modification and strong operability, so it has a very wide application prospect. The emergence of immune checkpoint inhibitor PD-L1 aptamer provides a new idea to improve the anti-cancer effect of collaborative therapy, and its adverse reactions are few, safe and controllable (Li et al., 2020b; Joo et al., 2020; Yuan et al., 2021).

Due to the particularity of TME, traditional drug carriers with passive diffusion are faced with the shortcomings of poor initiative and selectivity. Therefore, in addition to the enhanced permeability and retention (EPR) effect of solid tumors, nanodrive technology can also be used for autonomous movement to promote effective enrichment, retention and penetration of therapeutic drugs in the lesion site (Kwon et al., 2021; Mujtaba et al., 2021; Wu et al., 2022). The driving force types of nanodrive technology include biological drive, chemical drive, optical drive, ultrasonic drive, magnetic drive and electric drive (Li et al., 2022; Shi et al., 2022; Xie et al., 2022). The generation of these artificially driven devices has attracted extensive attention due to their potential applications in nanomachines, nanomedicine, nanoscale transport and assembly, nanorobots, fluid systems and chemical sensors (Han et al., 2021; Ji et al., 2021; Jiao et al., 2021; Xu et al., 2021; Zhang et al., 2022). Through intravenous injection or *in situ* injection, the biomimetic nanocomposite with driving function can effectively improve the diffusion and penetration of drugs in tumor tissues by using its autonomous movement ability, so as to achieve good therapeutic effect (Lyu et al., 2021). Recently, the use of enzymes as biocatalytic units to drive the movement of various nano-materials or cells has been reported, inspired by the natural properties of biological enzymes, mild reaction conditions, and non-toxicity of substrate fuels (Zheng et al., 2022). The biomimetic nanocomposites driven by hydrogen peroxide have great value in the treatment of blood diseases (Zhang et al., 2019b). Catalase has been used in many studies as an attractive reagent to break down excess hydrogen peroxide in TME into oxygen. Oxygen can not only be used as propellant to drive nanomaterials forward, but also as oxygen supplement to relieve tumor hypoxia (Liu et al., 2016). In particular, in order to reduce the toxic side effects of treatment, biomimetic nanocomposites with autonomous locomotion ability should have the ability to actively target target cells and diseased tissues.

**FIGURE 1:**
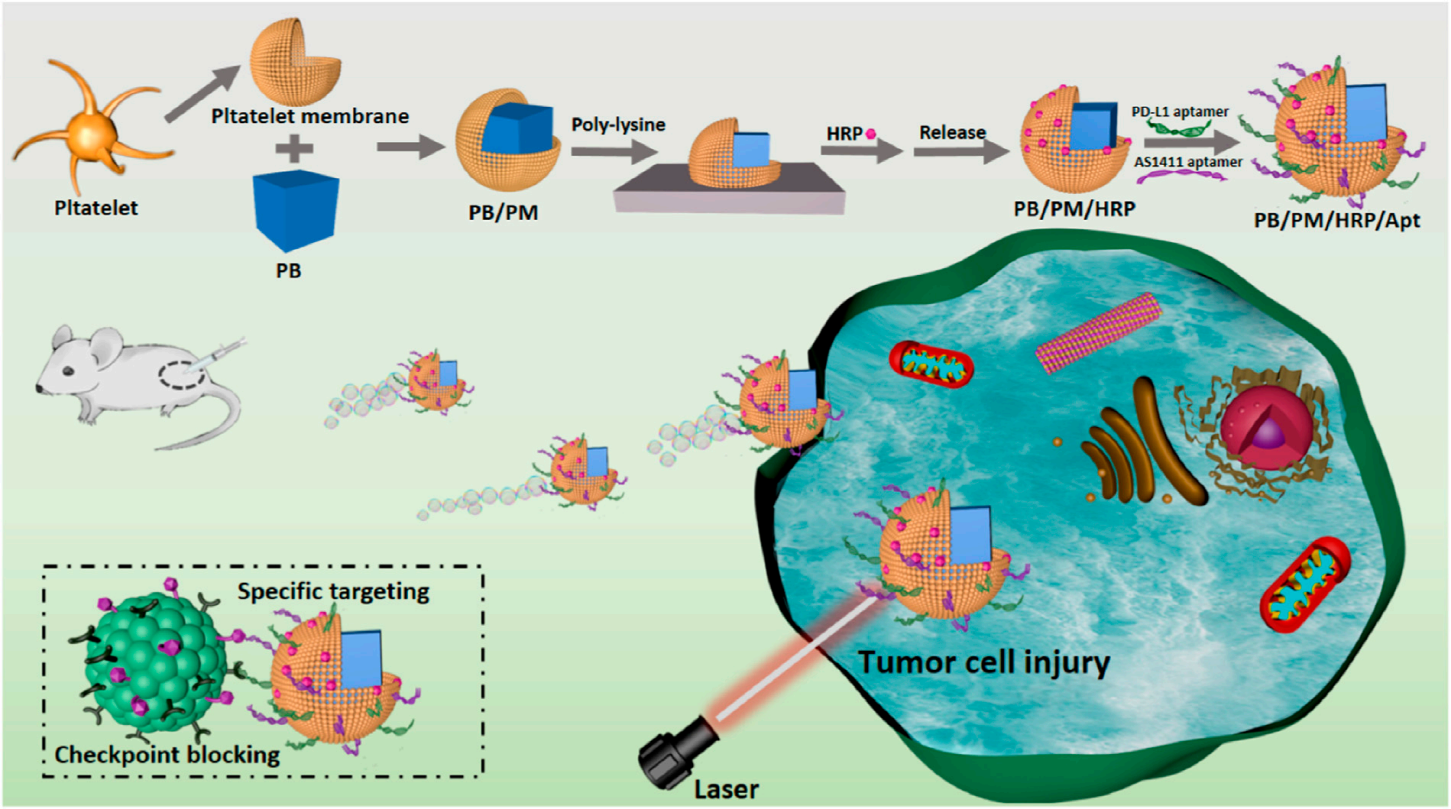
Schematic diagram of the synthesis and preparation process of PB/PM/HRP/Apt biomimetic nanocomposite and the mechanism of collaborative therapy.

Here, we have designed and synthesized Prussian blue biomimetic nanocomposites considering the above problems in current cancer treatment and the actual needs of disease treatment. First, we combined PBs with good photothermal conversion efficiency with PM to protect nanomaterials from immune clearance. The ability of PB/PM to specifically target cancer cells and inflammatory sites can effectively enhance the aggregation of PB at tumor sites, obtain good photothermal properties under near-infrared light and induce immune responses. Then, the surface of the synthesized biomimetic nanocomposite was modified with horseradish peroxidase (HRP). The distribution of HRP on the surface of the biomimetic nanocomposite will lead to the decomposition of excess hydrogen peroxide in TEM, and generate a chemical driving effect to enhance the penetration and accumulation of nanocomposite in tumor cells. Finally, to achieve immunotherapy and enhanced targeting, the surface of the synthesized biomimetic nanocomposite was modified with the immune checkpoint inhibitor PD-L1 aptamer and the AS1411 aptamer. PB/PM/HRP/Apt biomimetic nanoplatform combines a variety of therapeutic methods to greatly enhance the anti-cancer effect (Figure 1).

## 2 Experimental section

### 2.1 Synthesis of PB

At 25°C, 3 g polyvinylpyrrolidone (PVP) was put into a 250 ml three-neck flask with 80 mL deionized water and dissolved under continuous agitation. Next, 264 mg K_3_ [Fe(CN)_6_] and HCl (0.8 mL, 0.1 mol/L) were added under continuous agitation, and the mixture was evenly mixed for 30 min. Then the stirred products were put into an oil bath with an oil temperature of 80°C. PBs can be obtained after 20 h of heating without stirring in a constant temperature oil bath. To purify the prepared PBs, the product was washed three times with deionized water after centrifugation and washing twice with absolute ethanol. Finally, the purified PBs was dispersed in 10 ml of PBS and stored sealed at 4°C.

### 2.2 Synthesis of PB/PM

The 120 μl PM solution was mixed with 100 μL PB (0.5 mg/ml), and the resulting mixture was sonicated for 35 min to obtain PB/PM. In order to maintain the activity of membrane proteins, ice was added during the sonication process. Finally, the newly prepared PB/PM was placed in PBS buffer at 4°C overnight. Then, after centrifugation at 8000 rpm for 6 min, the supernatant was discarded and the precipitate was dissolved in PBS solution. This procedure was repeated three times to remove excess cell membrane, resulting in purified PB/PM, which was dispersed in 1 ml PBS solution and set aside.

### 2.3 Synthesis of PB/PM/HRP/Apt

PB/PM/HRP/Apt was fabricated on a 12-well plate modified from commercial polylysine plate (PLL). To complete the surface modification, PB/PM (0.5 mg/ml) was first centrifuged at 900 rpm for 3 min and incubated for 1 h. Then, 50 µl HRP-Bio solution (0.5 mg/ml), 10 µl EDC solution (0.5 M) and 10 µl NHS solution (0.5 M) were added to the reaction system at the same time and incubated at 25°C for 2 h. The incubation needs to be washed three times with PBS after completion to remove the free agent between each reaction. The products were evenly mixed with 50 µl FITC-Avi solution (0.5 mg/ml) and incubated at 25°C for 1 h. Next, 50 µl PD-L1 aptamer solution (10 μmol/ml), 10 µl EDC solution, and 10 µl NHS solution were mixed for 25 min to activate the carboxyl group. The activated PD-L1 aptamer solution was transferred to a centrifuge tube containing PB/PM/HRP and incubated for 4 h. PB/PM/HRP/Apt was obtained and stored at 4°C. PB/PM/HRP/Apt was obtained and stored at 4°C.

### 2.4 Photothermal efficiency testing of materials

The power of 808 nm NIR laser was set to 2 W cm^−2^, and PB and PB/PM/HRP/Apt dispersions in centrifuge tubes were irradiated with NIR laser for 10 min. The infrared thermal imager was used to collect images at different time points, and the temperature curve of different time periods was obtained by data processing system.

### 2.5 Cell internalization assay

After growing 4T1 and L02 cells in confocal dishes for 12 h, 400 μg/ml PB/PM/HRP/Apt was added to the cells and incubated for 2 h to study the intracellular uptake of PB/PM/HRP/Apt biomimetic nanocomplexes. After incubation, the cells were thoroughly washed three times with PBS. 4T1 and L02 cells cultured in confocal dishes were stained with Hoechst 33342 dye for 15 min, respectively. Finally, the internalization of PB/PM/HRP/Apt in cells was observed by confocal microscopy.

### 2.6 Cytotoxicity assay

To distinguish between living and dead cells, 10^4^ 4T1 cells were poured into small petri dishes and cultured for 12 h, and then the different treatment groups were added to the small petri dishes separately. After incubation for a certain time, the supernatant was absorbed into the centrifuge tube, nitrated with trypsin, and all cells were collected by adding fresh medium and combined into the same centrifuge tube. After centrifugation, cells were washed with PBS and stained with propidium iodide (PI, 1 μl) and Calcein AM (1 μl) for 30 min. Finally, we used a TCS SP8 II laser confocal microscope for observation and photography.

## 3 Results and discussion

### 3.1 Synthesis and characterization of PB/PM/HRP/Apt

**FIGURE 2:**
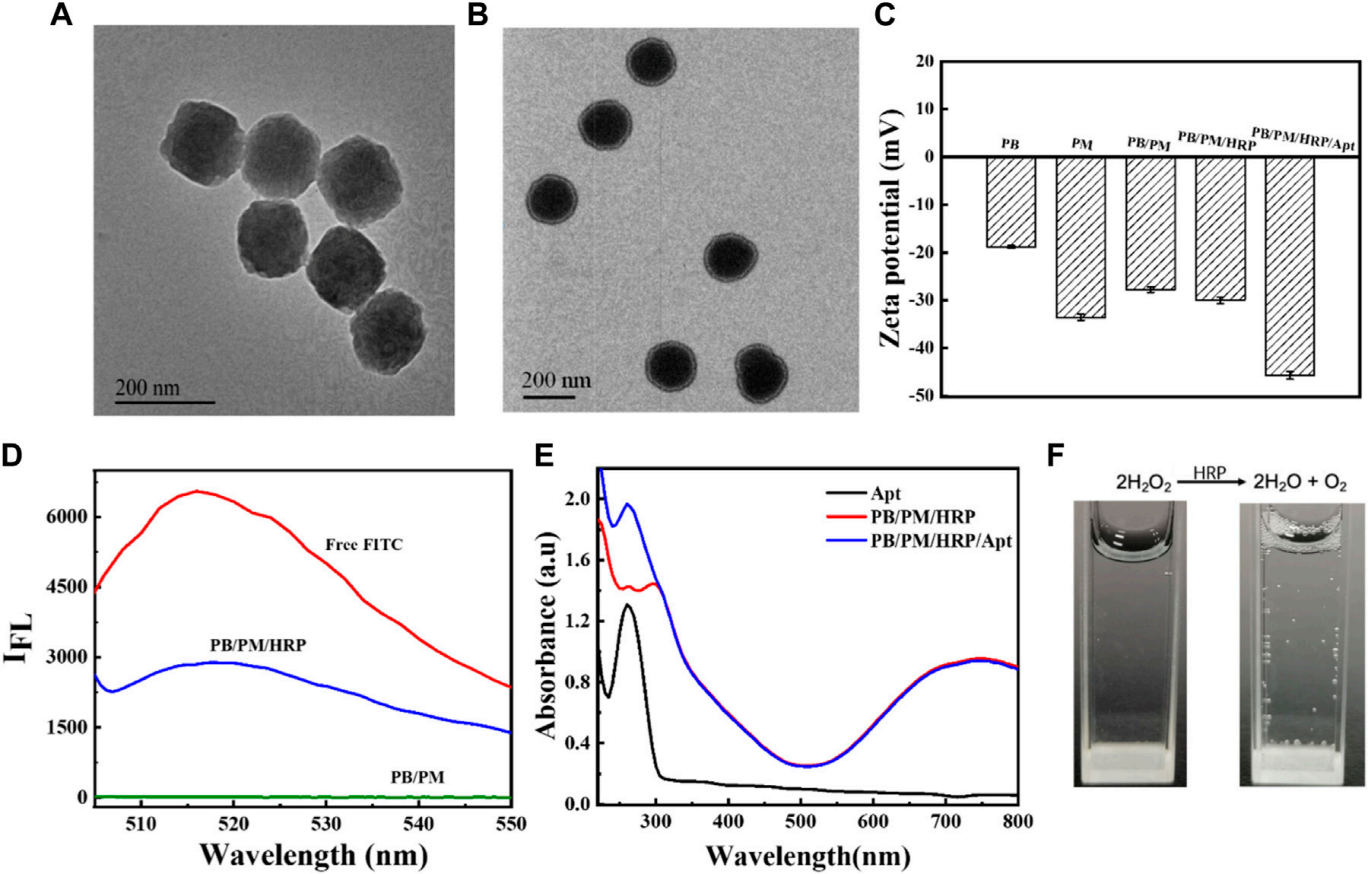
Synthesis and characterization of PB/PM/HRP/Apt. **(A)** TEM image of PB. **(B)** TEM image of PB/PM. **(C)** Zeta potential changes of PB, PM, PB/PM, PB/PM/HRP, PB/PM/HRP/Apt. **(D)** Fluorescence spectra of free FITC, PB/PM/HRP and PB/PM. **(E)** UV-Vis absorption spectra of Apt, PB/PM and PB/PM/Apt. **(F)** Reaction cup evolution of bubbles in hydrogen peroxide solution, add PB/PM/Apt reagent on the left and PB/PM/HRP/Apt reagent on the right.

In acidic environments, [Fe(CN)_6_]^3-^ as a precursor and polyvinylpyrrolidone (PVP) as a protective agent can slowly release ferrous ions and be oxidized to iron ions. The formed iron ions can react with the undecomposed ions to form PBs. Because of the slow reaction process and high monodispersity of NPs, this method is considered to be the best method for PBs preparation (Supplementary Figure S1). On the basis of PBs synthesis, PB/PM was obtained by ultrasonic method. The amino groups on the PMs lay the foundation for further binding with HRP and aptamers. Then, using EDC and NHS as activators, HRP modified with FITC fluorophore was fixed on the surface of PB/PM. Finally, the carboxyl modified PD-L1 aptamer and AS1411 aptamer were successively introduced to obtain the biomimetic nanocomposite PB/PM/HRP/Apt. Using transmission electron microscopy (TEM) images, we found that PBs was uniformly distributed and uniform in size, with an average size of about 184 nm (Figure 2A). In addition, an obvious core-shell structure can be seen in the TEM image of PB/PM, with an average size of about 200 nm and a thickness of about 9 nm (Figure 2B). To further validate the successful encapsulation of PB/PM/HRP/Apt, we used dynamic light scattering (DLS) analysis to determine particle size and Zeta potential. Consistent with the data obtained by TEM, the hydrodynamic diameter of PB/PM nanoparticles was increased to 196 nm, and after modification of HRP and aptamer, the hydrodynamic diameter of PB/PM/HRP/Apt biomimetic nanocomposite was increased to 226 nm (Supplementary Figure S2). In addition, the Zeta potential of PBs was −24.2 mV. After wrapping the PM, the potential decreased to −27.8 mV under the influence of the PM, and the potential of the final PB/PM/HRP/Apt biomimetic nanocomposite was −45.38 mV (Figure 2C).

Next, sodium dodecyl sulfate polyacrylamide gel electrophoresis (SDS-PAGE) was used to observe the protein bands of PB, PMs and PB/PM, so as to determine whether the platelet membrane was successfully coated with PBs. As shown in Supplementary Figure S3, PB/PM and PM showed similar protein profiles, indicating that the protein had been completely retained in PB/PM. Meanwhile, the successful preparation of PBs and PB/PM/HRP/Apt was demonstrated by ultraviolet-visible spectroscopy (UV-Vis). PB has a strong absorbance in the range of 700–900 nm, which is the characteristic peak of PB, which proves that PB synthesis is successful. The absorbance values of PBs at different concentrations were different, and the absorbance values of PBs increased with the increase of concentration. Moreover, the absorption peak of PB at 700–900 nm also indicates that PB can convert light energy into heat energy under laser irradiation at 808 nm, which can be used for subsequent PTT (Supplementary Figure S4). In addition, PB/PM/HRP is fluorescent because the surface of HRP is modified by the fluorophore FITC. In the fluorescence spectrum, the fluorescence signal of FITC was detected in PB/PM/HRP, but not in PB/PM without HRP modification, indicating that the biomimetic nanocomposite successfully achieved HRP loading (Figure 2D). PB/PM/HRP/Apt shows a UV absorption peak at 260 nm, indicating the successful loading of PD-L1 aptamer and AS1411 aptamer (Figure 2E). Finally, the prepared PB/PM/HRP/Apt was dissolved in hydrogen peroxide solution, and gas emission was seen (Figure 2F). This indicates that HRP, when hydrogen peroxide is used as a fuel, will decompose hydrogen peroxide to produce oxygen, so as to convert chemical energy into its own driving force at the micro and nano scale. The above results indicated that PB/PM/HRP/Apt was successfully prepared.

### 3.2 PB/PM/HRP/Apt photothermal performance evaluation

**FIGURE 3:**
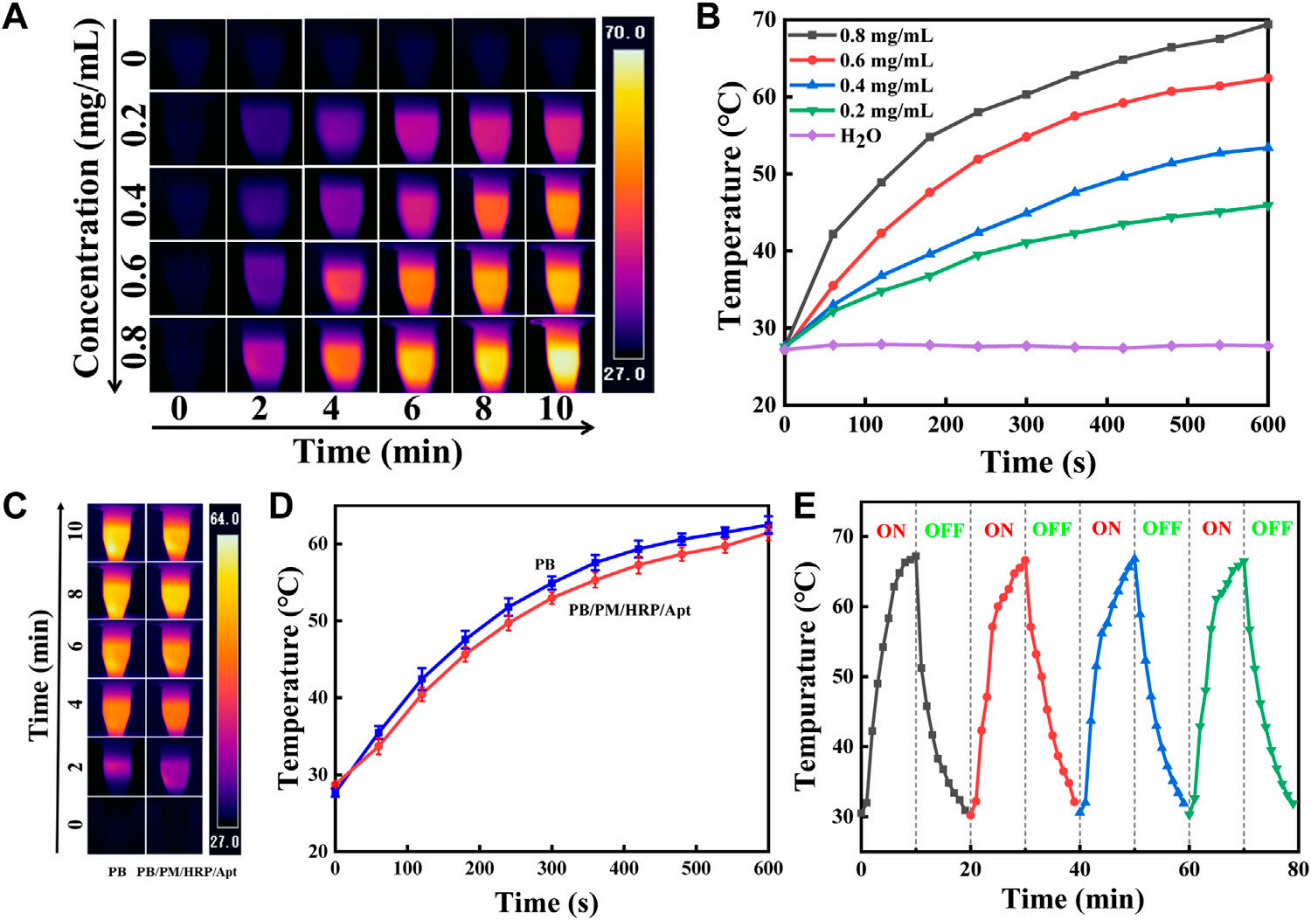
Characterization of photothermal properties of PB/PM/HRP/Apt. **(A)** Infrared thermal images of PB/PM/HRP/Apt at different concentrations (0–0.8 mg/ml, 2 W cm^−2^). **(B)** Concentration dependence curve of PB/PM/HRP/Apt (0–0.8 mg/ml, 2 W cm^−2^). **(C)** Infrared thermogram of PB and PB/PM/HRP/Apt exposed to 808 nm laser for 10 min (0.6 mg/ml, 2 W cm^−2^). **(D)** Temperature rise curve of PB and PB/PM/HRP/Apt (0.6 mg/ml, 2 W cm^−2^). **(E)** Temperature variation of PB/PM/HRP/Apt under repeated laser irradiation for four consecutive heating-cooling cycles (10 min irradiation for each cycle).

The photothermal effect of PB/PM/HRP/Apt was studied by recording the temperature rise under 808 nm laser irradiation. It is observed that PB/PM/HRP/Apt has a significant concentration and irradiation time dependent photothermal effect. We set the power density of 808 nm NIR laser as 2 W cm^−2^, and irradiated PB/PM/HRP/Apt dispersions for 10 min continuously, and photographed the temperature changes in real time with infrared camera (Figure 3A). It can be seen that the temperature curve increases in a concentration dependent way when the concentration of PB/PM/HRP/Apt increases (Figure 3B), indicating that the temperature increase is positively correlated with the concentration of PB/PM/HRP/Apt. In particular, PB/PM/HRP/Apt at a concentration of 0.6 mg/ml can reach the optimal therapeutic temperature for the tumor after 3 min and kill the cancer cells by thermal ablation.

Meanwhile, under the same power density, the dispersing solution of PB/PM/HRP/Apt and PBs were irradiated for 10 min respectively. After 10 min, the temperature of PB/PM/HRP/Apt increased to about 62°C and that of PB/PM/HRP/Apt increased to 61°C, with little difference between them. And gradually become stable (Figure 3D). These results show that the modified PBs still have high photothermal conversion performance and have little effect on their own photothermal effect. To investigate photothermal stability, the NIR laser was periodically exposed during each cycle and subjected to 10 min of effective irradiation (Figure 3E). After four radiation cycles (10 min each), the maximum temperature of PB/PM/HRP/Apt did not decrease significantly, indicating that PB/PM/HRP/Apt had excellent photothermal stability. In conclusion, PB/PM/HRP/Apt has obvious photothermal conversion efficiency and can be used as a photothermal reagent to treat tumors by producing local high temperature, which provides a guarantee for the application of PB/PM/HRP/Apt in cancer treatment.

### 3.3 Cellular uptake and internalization

**FIGURE 4:**
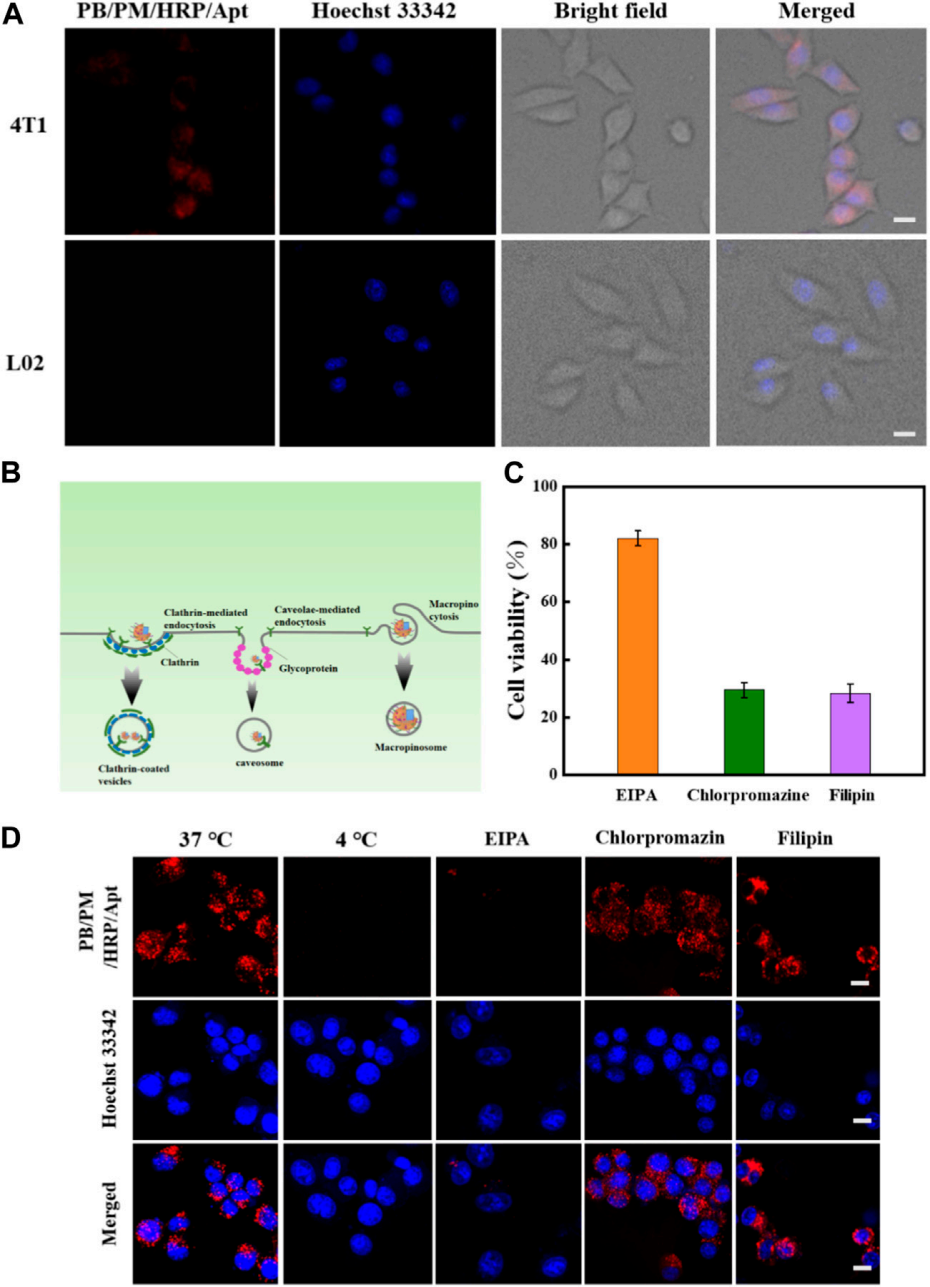
*In vitro* study of endocytosis mechanism of PB/PM/HRP/Apt. **(A)** Cellular internalization of PB/PM/HRP/Apt on 4T1 cells and L02 cells. **(B)** Schematic representation of clathrin-mediated endocytic pathway, caveolae mediated endocytic pathway, and macropinocytosis. **(C)** Cytotoxicity data after adding different inhibitors. **(D)** CLSM images of PB/PM/HRP/Apt in 4T1 cells pretreated with various endocytosis inhibitors (EIPA, Chlorprozine, Filipin) and at 37°C or 4°C. Scale bar: 10 μm.

Effective treatment requires efficient delivery of drugs to cancer cells. To demonstrate that PB/PM/HRP/Apt can be selectively internalized by tumor cells, we used 4T1 cells, L02 cells and MCF-7 cells to study the cellular internalization ability of PB/PM/HRP/Apt. The PB/PM/HRP/Apt biomimetic nanocomposite was incubated with the cells for 2 h, and then the PB/PM/HRP/Apt that did not enter the cells was washed with PBS buffer. Cell internalization was observed by confocal laser scanning microscope (CLSM) after incubation with nuclear fluorescent dye Hoechst 33342 in the incubator for 15 min. Through the bright field, fluorescence image and superposition image of CLSM, we can clearly see that the nucleus of 4T1 cells appears blue fluorescence, and the cytoplasm appears red fluorescence. These results indicated that PB/PM/HRP/Apt was successfully entered the cytoplasm of 4T1 cells. In contrast, no significant PB/PM/HRP/Apt fluorescence signal was detected in L02 cells treated under the same conditions (Figure 4A). Moreover, no significant PB/PM/HRP/Apt fluorescence signal was detected in MCF-7 cells (Supplementary Figure S5). Next, fluorescence analysis was performed by flow cytometry to verify the absorption of composite nanomaterials PB/PM/HRP/Apt by 4T1 cells (Supplementary Figure S6). The results showed that after 4T1 cells were incubated with composite nanomaterials PB/PM/HRP/Apt, the peak position changed and the fluorescence intensity increased significantly. In order to explore the mechanism of PB/PM/HRP/Apt uptake in 4T1 cells, microplatereader and confocal laser scanning microscopy were used to study the mechanism. Three inhibitors were selected, namely EIPA, a macropinocytosis inhibitor, chlorpromazine, a clathrin pathway inhibitor, and filipin, a caveolae-mediated endocytosis pathway inhibitor. First, 4T1 cells were pretreated with three inhibitors. The pre-treated 4T1 cells were added with PB/PM/HRP/Apt and incubated for a certain time. The cells were irradiated with laser. The activity of 4T1 cells treated with EIPA was significantly higher than that treated with the other two groups of inhibitors, indicating that only a small amount of PB/PM/HRP/Apt entered the cells and could not play the anticancer function normally (Figure 4C). Next, the uptake of PB/PM/HRP/Apt in 4T1 cells incubated at 37°C or 4°C was compared. Confocal images showed that PB/PM/HRP/Apt uptake was normal at 37°C, while PB/PM/HRP/Apt uptake at 4T1 decreased significantly at 4°C. Since the fluidity of cell membrane decreases at 4°C, this will affect the entry of nanomaterials into cells. 4T1 cells were pretreated with EIPA, chlorpromazine and filipin at 37°C, and then cultured with PB/PM/HRP/Apt for a certain period of time. We found that the fluorescence content of PB/PM/HRP/Apt in 4T1 cells pretreated with EIPA was significantly reduced, while the fluorescence signal in the cytoplasm of 4T1 cells pretreated with chlorpromazine and filipin was still significant (Figure 4D). These results indicated that PB/PM/HRP/Apt entered 4T1 cells through macropinocytosis.

### 3.4 *In Vitro* antitumor efficacy

**FIGURE 5:**
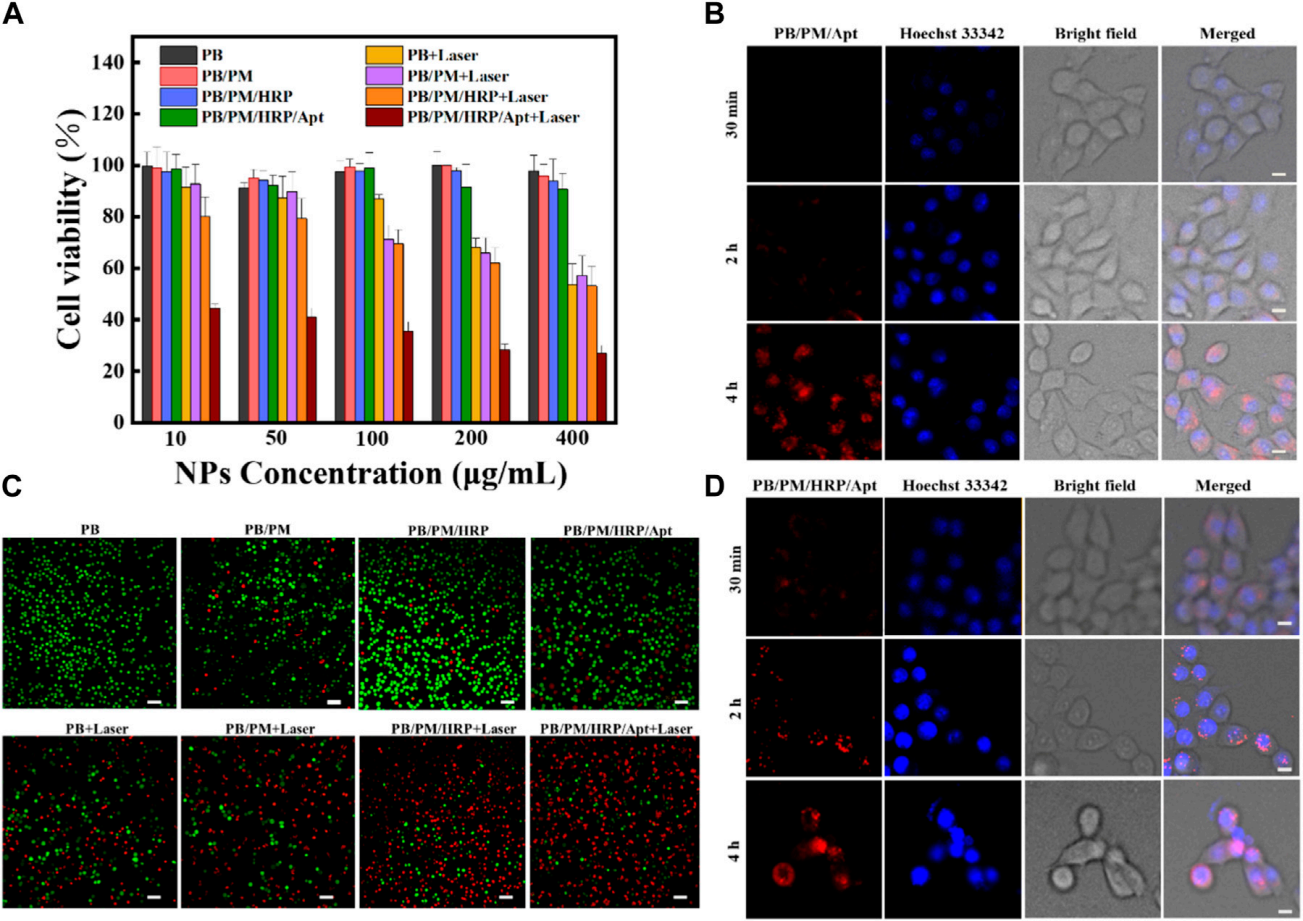
*In vitro* cytotoxicity assessment. **(A)** Viability of 4T1 cells treated with free PB, PB/PM, PB/PM/Apt, PB/PM/HRP/Apt and laser irradiation. **(B)** Confocal fluorescence images of 4T1 cell viability determined by different treatments. Scale bar: 50 μm. **(C)** Confocal fluorescence imaging of 4T1 cells treated with PB/PM/Apt at different incubation times. Scale bar: 10 μm. **(D)** Confocal fluorescence imaging of 4T1 cells treated with PB/PM/HRP/Apt at different incubation times. Scale bar: 10 μm.

To further investigate the cytotoxicity and biosafety of the biomimetic nanocomposites, we investigated the efficacy of PB/PM/HRP/Apt combined therapy using 4T1 cells. Firstly, CCK-8 assay was used to detect the cell viability of 4T1 after different treatments. 4T1 cells were treated with PB, PB/PM, PB/PM/Apt, PB/PM/HRP/Apt at different concentrations (PB: 400, 200, 100, 50, 10 μg/mL) for a certain time, and the survival rate of cells was determined. As shown in Figure 5A, the survival rate of 4T1 cells treated with PB, PB/PM, PB/PM/Apt and PB/PM/HRP/Apt was about 90%, and the cytotoxicity was almost negligible, which indicated that the PB/PM/HRP/Apt prepared by us had good biocompatibility. In addition, the survival rate of PB/PM-treated cells after NIR laser irradiation was significantly lower than that of PB + laser treated cells, indicating that the specific targeting ability of PM enhances its killing ability on tumor cells. The survival rate of PB/PM/HRP + laser group was lower than that of PB/PM + laser alone group, which may be because the effective movement of PB/PM/HRP under fuel can effectively improve the binding efficiency of PB/PM/HRP to biological targets. In the PB/PM/HRP/Apt group, the survival rate of 4T1 cells irradiated with 808 nm NIR laser was 26.32%. This result indicates that the targeting effect of AS1411 aptamer increases the internalization effect of cancer cells on the biomimetic nanocomposite, thus improving the therapeutic effect. Next, we further studied the synergistic effect of PB/PM/HRP/Apt. 4T1 cells were stained with Calcein AM and PI to obtain CLSM images of live (green) and dead (red) cells (Figure 5B), which were in general agreement with CCK-8 experiments. It can be seen that PB/PM/HRP/Apt can play a powerful killing effect on cancer cells under laser irradiation.

From the above results, it can be concluded that PB/PM/HRP/Apt + Laser has the strongest ability to induce related apoptosis of 4T1 cells. In addition, in order to determine the application potential of HRP in practical scenarios, PB/PM/Apt and PB/PM/HRP/Apt were incubated with 4T1 cells for different times, which proved that PB/PM/HRP/Apt had good exercise ability (Figures 5C, D). CLSM images showed that in the presence of hydrogen peroxide, the red fluorescence of 4T1 cells in the PB/PM/HRP/Apt group was significantly higher than that in the PB/PM/Apt group at 30 min and 2 h, indicating that the promoting effect of PB/PM/HRP/Apt could successfully enhance the internalization of cancer cells to nanomaterials. The accumulation effect tends to be the same at 4 h, indicating that PB/PM/HRP/Apt can achieve better cancer treatment effect in a certain period of time.

### 3.5 *In Vivo* antitumor efficacy

**FIGURE 6:**
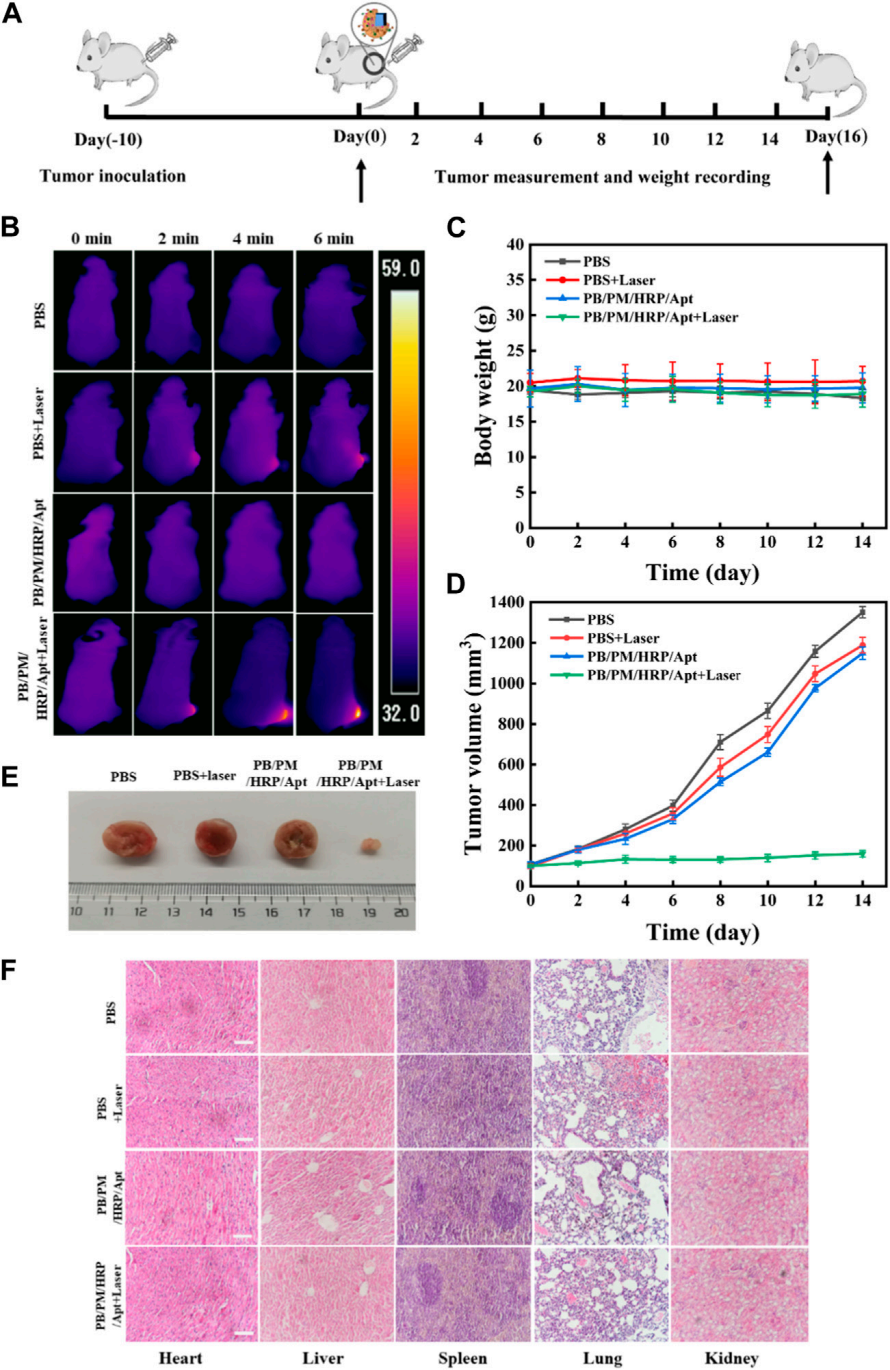
Anticancer effect of PB/PM/HRP/Apt *in vivo*. **(A)** Schematic representation of tumor formation and treatment outcome in 4T1 tumor-bearing mice. **(B)**Temperature changes of tumor-bearing mice. **(C)** Curve of weight change. **(D)** The corresponding tumor growth curves of 4T1 solid tumor-bearing mice monitored every 2 days after different treatments, and the data are expressed as mean values (n = 5). **(E)** Representative digital photographs of dissected tumors in each group after treatment. **(F)** Histological analysis of major organs was performed by H&E staining. Scale bar: 100 μm.

After confirming that PB/PM/HRP/Apt + laser has good photothermal characteristics, targeting, and anti-tumor effect *in vitro*, we then tested the anti-tumor effect of biomimetic nanocomposite in 4T1 tumor bearing mouse model (Figure 6A). First, 10 days after subcutaneous injection of 4T1 cells into BALB/c Nude mice, tumors grew to 100–150 mm^3^. Then we divided the mice with 4T1 tumor into four groups for orthotopic injection. The four groups of mice were treated with PBS, PBS + laser, PB/PM/HRP/Apt and PB/PM/HRP/Apt + laser, respectively. To examine the tumor targeting and aggregation ability of PB/PM/HRP/Apt against cancer cells, we performed photothermal imaging using 4T1 tumor-bearing mice, followed by measuring the temperature of the tumor surface with an infrared camera. As shown in Figure 6B, it can be seen that there was no significant change in temperature in the tumor area of the mice injected with PBS. Another group of PBS-treated mice treated with an 808 nm laser showed only a slight increase in tumor temperature. In mice injected with PB/PM/HRP/Apt, the temperature in the irradiated area rose rapidly to 58.2°C (Supplementary Figure S7). It has been reported that tumor tissue is less heat tolerant than normal tissue. Selective necrosis occurs when the temperature of the tumor tissue is higher than the local temperature. Therefore, PB/PM/HRP/Apt irradiated by laser have the ability to induce photothermal ablation of cancer cells. After verifying the photothermal conversion ability of PB/PM/HRP/Apt in a mouse tumor model, we next evaluated the antitumor effect of PB/PM/HRP/Apt by measuring body weight and tumor volume in mice. The volume and body weight of each group of mice were measured every 2 days throughout the *in vivo* experiment, as shown in Figure 6C. Obviously, the body weight of mice in each treatment group did not change significantly, indicating that PB/PM/HRP/Apt biomimetic nanocomposite has good biocompatibility. In the curve of tumor volume change in mice, it can be seen that the tumor grew rapidly in the PBS group because PBS buffer could not treat the tumor. As in the PBS group, the tumor volume of mice injected with PBS + laser and PB/PM/HRP/Apt also showed a strong upward trend. However, compared with the other groups, the tumor growth rate of the mice injected with PB/PM/HRP/Apt + laser was much slower and showed a downward trend (Figure 6D). This is amply demonstrated by the fact that PB/PM/HRP/Apt produces strong photothermal responses in mice and highlights the therapeutic advantages of immunotherapy and chemical drive.

To elucidate an effective long-term immune memory response, we excised tumors in the PB/PM/HRP/Apt group and primed mice again 1 week later with 3×10^5^ cells. PB/PM/HRP/Apt group significantly inhibited tumor growth, and no tumor was found 21 days after restimulation. Next, we evaluated the cytokine level by enzyme-linked immunosorbent assay (ELISA) to determine whether the synergistic effect of PTT and immune checkpoint inhibitor caused strong immune response. Cytokines can promote cell-cell interactions and play an important role in immune response to tumor growth. We found that the contents of interferon-γ (IFN-γ), tumor necrosis factor (TNF-α), granzyme B and interleukin-6 (IL-6) in the tumor of mice treated with PB/PM/HRP/Apt + laser were significantly higher than those of other groups (Supplementary Figure S8). We all know that IFN-γ and granzyme B, as a macrophage factor, not only promotes inflammatory response but also has antitumor activity. In addition, TNF-α and IL-6, as a proinflammatory factor, plays an important role in tumor necrosis. These results indicate that synergic PTT combined with PD-L1 aptamer can trigger a strong immune response, inhibit tumor recurrence and metastasis, and prolong the survival time of mice. At the end of the treatment cycle, the mice were dissected, and the heart, liver, spleen, lung, kidney and tumor were extracted, and the *in vitro* images of mouse tumors were taken (Figure 6E). To evaluate the efficacy of different treatment groups, hematoxylin-eosin (H&E) staining was performed on treated tumor tissues. Staining showed that PTT, immunotherapy combined with chemical-driven PB/PM/HRP/Apt + laser group had the best therapeutic effect, and a large number of necrotic cancer cells and inflammatory cells were found in H&E sections. However, the necrosis of cancer cells in the PBS group, PB/PM/HRP/Apt and PBS + laser groups was not obvious (Supplementary Figure S9). At present, biomedical nanomaterials have some problems, such as poor biological metabolism and systemic toxicity. To evaluate the safety and biocompatibility of PB/PM/HRP/Apt biomimetic nanocomposites *in vivo*, H&E staining was performed on the heart, liver, spleen, lung and kidney of treated mice (Figure 6F). There was no significant difference in H&E-stained organ sections among groups, and no obvious injury or inflammation was found in each tissue. These results indicate that the designed nanocomposite not only has good anticancer activity, but also has good biocompatibility.

## 4 Conclusion

In conclusion, inspired by the inherent tumor homing ability and excellent biocompatibility of platelets, we have developed biomimetic nanocomposites to achieve a combination therapy combining photothermal therapy, anti-PD-L1 immunotherapy, and chemical drive. The PB surface with good photothermal properties was functionalized, coated with PMs with cancer cell targeting and immune escape ability, modified with HRP on PB/PM, and finally modified with AS1411 aptamer and PD-L1 aptamer. The obtained PB/PM/HRP/Apt can more easily evade immune clearance and target tumor tissues, and this specific targeting method can significantly enhance the accumulation of PB/PM/HRP/Apt in tumor sites. Our PD-L1 aptamer is used to block the immunosuppressive pathway, enhance anti-tumor immunity, and induce immune response. By decomposing hydrogen peroxide, the biomimetic nanocomposite can move effectively at micro-nano scale. In this way, the biomimetic nanocomposite PB/PM/HRP/Apt can achieve the comprehensive therapeutic effect driven by photothermal therapy, immunotherapy and enzyme chemistry, which provides a broad application prospect for the flexible combination of multiple therapeutic methods in the biomedical field.

## Data Availability

The original contributions presented in the study are included in the article/Supplementary Material, further inquiries can be directed to the corresponding author.

## References

[B1] Boehnke N. Straehla J. P. Safford H. C. Kocak M. Rees M. G. Ronan M. (2022). Massively parallel pooled screening reveals genomic determinants of nanoparticle delivery. Science 377 (6604), eabm5551. eabm5551. 10.1126/science.abm5551 35862544 PMC10249039

[B2] Chen C. L. Song M. Y. Du Y. Y. Yu Y. Li C. G. Han Y. (2021). Tumor-associated-macrophage-membrane-coated nanoparticles for improved photodynamic immunotherapy. Nano Lett. 21 (13), 5522–5531. 10.1021/acs.nanolett.1c00818 34133181

[B3] Guo Y. Cao X. Zhang S. (2021a). Au-Fe_3_O_4_ nanoagent coated cell membrane for targeted delivery and enhanced chem/photo therapy. Chem. Commun. 57 (81), 10504–10507. 10.1039/d1cc03454e 34528033

[B4] Guo Y. Li W. Liu S. Jing D. Wang Y. Feng Q. (2022). Construction of nanocarriers based on endogenous cell membrane and their application in nanomedicine. Chin. J. Chem. 40 (13), 1623–1640. 10.1002/cjoc.202100946

[B5] Guo Y. Liu F. Hu Y. Zheng X. Cao X. Zhu Y. (2020). Activated plasmonic nanoaggregates for dark-field *in situ* imaging for HER2 protein imaging on cell surfaces. Bioconjug. Chem. 31 (3), 631–638. 10.1021/acs.bioconjchem.9b00787 31944094

[B6] Guo Y. S. Zheng X. F. Gai T. T. Wei Z. Y. Zhang S. S. (2021c). Co-biomembrane-coated Fe_3_O_4_/MnO_2_ multifunctional nanoparticles for targeted delivery and enhanced chemodynamic/photothermal/chemo therapy. Chem. Commun. 57 (47), 5754–5757. 10.1039/d1cc01375k 34036980

[B7] Guo Y. Wu D. Zhang X. Zhang K. Luo Y. (2021b). Biomolecules in cell-derived extracellular vesicle chariots as warriors to repair damaged tissues. Nanoscale 13 (38), 16017–16033. 10.1039/d1nr04999b 34570853

[B8] Han X. Ding S. Fan L. Zhou Y. Wang S. (2021). Janus biocomposite aerogels constituted of cellulose nanofibrils and MXenes for application as single-module solar-driven interfacial evaporators. J. Mat. Chem. A 9 (34), 18614–18622. 10.1039/d1ta04991g

[B9] Ji X. Yang H. Liu W. Ma Y. Wu J. Zong X. (2021). Multifunctional parachute-like nanomotors for enhanced skin penetration and synergistic antifungal therapy. ACS Nano 15 (9), 14218–14228. 10.1021/acsnano.1c01379 34435494

[B10] Jiao C. Zhao C. Ma Y. Yang W. (2021). A Versatile strategy to coat individual cell with fully/partially covered shell for preparation of self-propelling living cells. ACS Nano 15 (10), 15920–15929. 10.1021/acsnano.1c03896 34591443

[B11] Joo K. I. Jeong Y. Hwang S. M. Shin M. Lee J. Sharma G. (2020). Harnessing the bioresponsive adhesion of immuno-bioglue for enhanced local immune checkpoint blockade therapy. Biomaterials 263, 120380. 10.1016/j.biomaterials.2020.120380 32942128

[B12] Kong F. He H. Bai H. Yang F. Ma M. Gu N. (2022). A biomimetic nanocomposite with enzyme-like activities and CXCR4 antagonism efficiently enhances the therapeutic efficacy of acute myeloid leukemia. Bioact. Mat. 18, 526–538. 10.1016/j.bioactmat.2022.03.022 PMC8976099 35415298

[B13] Kwon T. Kumari N. Kumar A. Lim J. Son C. Y. Lee I. S. (2021). Au/Pt-Egg-in-Nest nanomotor for glucose-powered catalytic motion and enhanced molecular transport to living cells. Angew. Chem. Int. Ed. 60 (32), 17579–17586. 10.1002/anie.202103827 34107153

[B14] Li D. Bao A. Chen X. Li S. Wang T. Zhang L. (2020a). Prussian blue@polyacrylic acid/Au aggregate Janus nanoparticles for CT imaging‐guided chemotherapy and enhanced photothermal therapy. Adv. Ther. 3 (10), 2000091. 10.1002/adtp.202000091

[B15] Li Q. Liu L. Huo H. Su L. Wu Y. Lin H. (2022). Nanosized Janus AuNR-Pt motor for enhancing NIR-II photoacoustic imaging of deep tumor and Pt(^2+^) ion-based chemotherapy. ACS Nano 16, 7947–7960. 10.1021/acsnano.2c00732 35536639

[B16] Li Y. H. Zhang X. Wan X. Y. Liu X. H. Pan W. Li N. (2020b). Inducing endoplasmic reticulum stress to expose immunogens: A dna tetrahedron nanoregulator for enhanced immunotherapy. Adv. Funct. Mat. 30 (48), 2000532. 10.1002/adfm.202000532

[B17] Liu B. Song Y. Liu D. (2017). Recent development in clinical applications of PD-1 and PD-L1 antibodies for cancer immunotherapy. J. Hematol. Oncol. 10 (1), 174. 10.1186/s13045-017-0541-9 29195503 PMC5712158

[B18] Liu M. Sun Y. Wang T. Ye Z. Zhang H. Dong B. (2016). A biodegradable, all-polymer micromotor for gas sensing applications. J. Mat. Chem. C 4 (25), 5945–5952. 10.1039/c6tc00971a

[B19] Lyu X. Liu X. Zhou C. Duan S. Xu P. Dai J. (2021). Active, yet little mobility: Asymmetric decomposition of H_2_O_2_ is not sufficient in propelling catalytic micromotors. J. Am. Chem. Soc. 143 (31), 12154–12164. 10.1021/jacs.1c04501 34339185

[B20] Mujtaba J. Liu J. Dey K. K. Li T. Chakraborty R. Xu K. (2021). Micro-bio-chemo-mechanical-systems: Micromotors, microfluidics, and nanozymes for biomedical applications. Adv. Mat. 33 (22), e2007465. 10.1002/adma.202007465 33893682

[B21] Qu Y. Chu B. Wei X. Chen Y. Yang Y. Hu D. (2021). Cancer-cell-biomimetic nanoparticles for targeted therapy of multiple myeloma based on bone marrow homing. Adv. Mat. 13, e2107883. 10.1002/adma.202107883 34877715

[B22] Shi J. Nie W. Zhao X. Yang X. Cheng H. Zhou T. (2022). An intracellular self-assembly-driven uninterrupted ROS generator augments 5-aminolevulinic-acid-based Tumor Therapy. Adv. Mat. 34 (30), e2201049. 10.1002/adma.202201049 35488781

[B23] Srivastava I. Xue R. Jones J. Rhee H. Flatt K. Gruev V. (2022). Biomimetic surface-enhanced Raman scattering nanoparticles with improved dispersibility, signal brightness, and tumor targeting functions. ACS Nano 16, 8051–8063. 10.1021/acsnano.2c01062 35471820

[B24] Wang S. Wang R. Meng N. Guo H. Wu S. Wang X. (2020). Platelet membrane-functionalized nanoparticles with improved targeting ability and lower hemorrhagic risk for thrombolysis therapy. J. Control. Release 328, 78–86. 10.1016/j.jconrel.2020.08.030 32853731

[B25] Wang T. He Z. Yuan C. S. Deng Z. W. Li F. Chen X. G. (2022). MMP-responsive transformation nanomaterials with IAP antagonist to boost immune checkpoint therapy. J. Control. Release 343, 765–776. 10.1016/j.jconrel.2022.02.018 35181414

[B26] Wu Z. Tang Y. Chen L. Liu L. Huo H. Ye J. (2022). *In-situ* assembly of Janus nanoprobe for cancer activated NIR-II photoacoustic imaging and enhanced photodynamic therapy. Anal. Chem. 94 (29), 10540–10548. 10.1021/acs.analchem.2c02108 35819004

[B27] Xie L. Liu T. He Y. Zeng J. Zhang W. Liang Q. (2022). Kinetics-regulated interfacial selective superassembly of asymmetric smart nanovehicles with tailored topological hollow architectures. Angew. Chem. Int. Ed. 61 (12), e202200240. 10.1002/anie.202200240 35085410

[B28] Xie W. Deng W. W. Zan M. Rao L. Yu G. T. Zhu D. M. (2019). Cancer cell membrane camouflaged nanoparticles to realize starvation therapy together with checkpoint blockades for enhancing cancer therapy. ACS Nano 13 (3), 2849–2857. 10.1021/acsnano.8b03788 30803232

[B29] Xu C. Wang S. Wang H. Liu K. Zhang S. Chen B. (2021). Magnesium-based micromotors as hydrogen generators for precise rheumatoid arthritis therapy. Nano Lett. 21 (5), 1982–1991. 10.1021/acs.nanolett.0c04438 33624495

[B30] Xue P. Yang R. Sun L. Li Q. Zhang L. Xu Z. (2018). Indocyanine green-conjugated magnetic prussian blue nanoparticles for synchronous photothermal/photodynamic tumor therapy. Nanomicro. Lett. 10 (4), 74. 10.1007/s40820-018-0227-z 30417006 PMC6208784

[B31] Yan S. Zeng X. Tang Y. Liu B. F. Wang Y. Liu X. (2019). Activating antitumor immunity and antimetastatic effect through polydopamine-encapsulated core-shell upconversion nanoparticles. Adv. Mat. 31 (46), e1905825. 10.1002/adma.201905825 31566283

[B32] Yang R. Hou M. Gao Y. Zhang L. Xu Z. Kang Y. (2019). Indocyanine green-modified hollow mesoporous Prussian blue nanoparticles loading doxorubicin for fluorescence-guided tri-modal combination therapy of cancer. Nanoscale 11 (12), 5717–5731. 10.1039/c8nr10430a 30865744

[B33] Yu H. Fan J. Shehla N. Qiu Y. Lin Y. Wang Z. (2021). Biomimetic hybrid membrane-coated xuetongsu assisted with laser irradiation for efficient rheumatoid arthritis therapy. ACS Nano 16, 502–521. 10.1021/acsnano.1c07556 34965104

[B34] Yuan K. Jurado-Sanchez B. Escarpa A. (2021). Dual-propelled lanbiotic based Janus micromotors for selective inactivation of bacterial biofilms. Angew. Chem. Int. Ed. 60 (9), 4915–4924. 10.1002/anie.202011617 33216439

[B35] Zhang C. Xia D. Liu J. Huo D. Jiang X. Hu Y. (2020a). Bypassing the immunosuppression of myeloid-derived suppressor cells by reversing tumor hypoxia using a platelet-inspired platform. Adv. Funct. Mat. 30 (22), 2000189. 10.1002/adfm.202000189

[B36] Zhang F. Lu G. Wen X. Li F. Ji X. Li Q. (2020b). Magnetic nanoparticles coated with polyphenols for spatio-temporally controlled cancer photothermal/immunotherapy. J. Control. Release 326, 131–139. 10.1016/j.jconrel.2020.06.015 32580043

[B37] Zhang K. Tu M. Gao W. Cai X. Song F. Chen Z. (2019a). Hollow prussian blue nanozymes drive neuroprotection against ischemic stroke via attenuating oxidative stress, counteracting inflammation, and suppressing cell apoptosis. Nano Lett. 19 (5), 2812–2823. 10.1021/acs.nanolett.8b04729 30908916

[B38] Zhang X. Chen C. Wu J. Ju H. (2019b). Bubble-propelled jellyfish-like micromotors for DNA sensing. ACS Appl. Mat. Interfaces 11 (14), 13581–13588. 10.1021/acsami.9b00605 30888785

[B39] Zhang Y. Yang F. Wei W. Wang Y. Yang S. Li J. (2022). Self-propelled Janus mesoporous micromotor for enhanced microRNA capture and amplified detection in complex biological samples. ACS Nano 16, 5587–5596. 10.1021/acsnano.1c10437 35357821

[B40] Zheng J. Qi R. Dai C. Li G. Sang M. (2022). Enzyme catalysis biomotor engineering of neutrophils for nanodrug delivery and cell-based thrombolytic therapy. ACS Nano 16 (2), 2330–2344. 10.1021/acsnano.1c08538 35138084

[B41] Zhou S. Zhen Z. Paschall A. V. Xue L. Yang X. Bebin-Blackwell A. G. (2021). FAP-targeted photodynamic therapy mediated by ferritin nanoparticles elicits an immune response against cancer cells and cancer associated fibroblasts. Adv. Funct. Mat. 31 (7), 2007017. 10.1002/adfm.202007017 PMC9273013 35822179

