# Supplementary material for: Self-actuated biomimetic nanocomposites for photothermal therapy and PD-L1 immunosuppression
Source: Front Chem. 2023 Mar 17;11:1167586. doi: 10.3389/fchem.2023.1167586 (PMC10063802; doi:10.3389/fchem.2023.1167586)
Supplement: Supplementary file 1 [file DataSheet1.pdf]

Supplementary Material

Self-actuated biomimetic nanocomposites for photothermal therapy and PD-L1 immunosuppression

Wenxin Li[1,2], Fen Li[1], Tao Li[1], Wenyue Zhang[1], Binglin Li[1], Kunrui Liu[1], Xiaoli Lun[1], Yingshu Guo[1,*]

*** Correspondence:** Corresponding Author: yingshug@126.com

[1]Shandong Provincial Key Laboratory of Molecular Engineering, School of Chemistry and Chemical Engineering, Qilu University of Technology (Shandong Academy of Sciences), Jinan, 250353, China

[2]Linyi University, Linyi, 276000, China

1 Materials and Methods

1.1 Materials and reagents

Polyvinylpyrrolidone (PVP) was purchased from Shanghai Yuanye Biotechnology Co., LTD. $K_3[Fe(CN)_6]$ was purchased from Shanghai MacLean Biochemical Technology Co., LTD. N-(3-dimethylaminopropyl)-n-ethyl aminopropyl diimide hydrochloride (EDC) and n-hydroxysuccinimide (NHS) were purchased from Shanghai Aladin Biochemical Technology Co., LTD. FITC-Avi and HRP-Bio were purchased from Shenggong Bioengineering (Shanghai) Co., LTD. PD-L1 aptamer (5'-COOH-ACGGGCCACATCAAC TCATTGATAGACAATGCGTCCACTGCCCGT-3') and AS1411 aptamer (5'-COOH-GGTGGTGGTGGTTGTGGTGGTGGTGG-3') Purchased from Zisheng Bioengineering (Shanghai) Co., LTD. Hoechst 33342 was provided by Beijing Solarbio Technology Co., LTD. Calcein AM/PI Cell activity and cytotoxicity assay kit was purchased from Shanghai Biyuntian Biotechnology Co., LTD. Cell Proliferation/Toxicity Assay Kit (CCK-8) was purchased from Dongren Chemical Technology (Shanghai) Co., LTD. Purchased 300 mesh copper mesh from ProSciTech. All other reagents are analytically pure and can be used directly without further purification. All animal experimental procedures and techniques were approved by the Animal Ethics Committee of Linyi University, and methods were carried out in accordance with the approved guidelines and laws.

1.2 The instrument.

The instrument. The product was characterized by transmission electron microscopy (TEM) (JEM-2100, JEOL). The fluorescence of the samples was measured using an F-4600 fluorescence spectrophotometer (Hitachi). The UV absorption spectra of the synthesized materials were measured using an Agilent UV-Vis spectrophotometer CARY-60. Mean particle size and Zeta potential were measured by dynamic light scattering (DLS), and confocal fluorescence imaging studies were performed using a laser scanning confocal microscope (LSCM) with an objective lens (×20) (Nikon C2 Plus). The cell viability assay was performed using BioTek Epoch full-wavelength microplate reader.

1.3 The preparation of the PM

10 mL of mouse whole blood was centrifuged at 1500 rpm for 10 min. The supernatant was separated into platelet-rich plasma (PRP). After PRP was centrifuged at 3000 rpm for 20 min, the precipitate was washed with PBS buffer and centrifuged repeatedly before platelets were obtained. Platelets were frozen at -80 °C and thawed at room temperature, and this process was repeated three times. Membranes were obtained by centrifugation at 8000 rpm for 10 min, washed with PBS containing protease inhibitors, and sonicated for 5 min. The resulting PM was dispersed in 2 mL of PBS and stored at -20 °C for later use.

1.4 Cell culture.

All 4T1 and L02 cells participating in the experiment were cultured at 37℃ in a constant temperature and humidity chamber with 95% humidity and 5% CO_2 concentration. The cell medium was DMEM containing 10% fetal bovine serum and 1% double antibody (penicillin-streptomycin).

1.5 CCK-8 analysis.

100 μL of 4T1 cell suspension at a concentration of 1×10^5 cells /mL was added to each well of a 96-well plate and precultured for 12 h in an incubator at 37 ° C. Then, 10 μL PB, PB/PM, PB/PM/Apt, PB/PM/HRP/Apt (PB: 400, 200, 100, 50, 10 μg/mL) were added to each well, and the incubation was continued for 4 h at 37℃. The supernatant was taken and 100 μL of fresh medium containing 10% CCK-8 solution was added to each well, taking care not to form bubbles in the Wells. The 96-well plates were incubated for an additional 35 min in an incubator, and finally the absorbance at 450 nm was determined using a microplate reader.

1.6 In vivo antitumor test.

All tumor-bearing mice were BALB/c nude mice aged 4-5 weeks. Each mouse was injected subcutaneously with 4T1 cells (3×10^7 cells/mouse). When the solid tumor size reached about 100 mm^3, the tumor-bearing mice were divided into four groups according to the experimental requirements. The four groups of mice were treated with PBS, PBS + laser, PB/PM/HRP/Apt and PB/PM/HRP/Apt + laser, respectively. Tumor volume and body weight were recorded every other day. During the treatment, the temperature changes and infrared thermal imaging images of tumor in each group were obtained by infrared thermal imager. At the end of the treatment cycle, the experimental mice were euthanized, and the tumor tissues and major organs of the treated mice were obtained. Finally, the tumor, heart, liver, spleen, lung, and kidney were analyzed by H&E staining.

2 Supplementary Figures

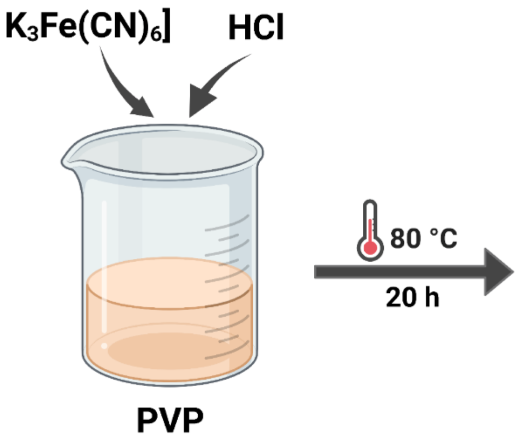

Supplementary Figure 1. Flow chart of hydrothermal synthesis of Prussian blue nanoparticles.

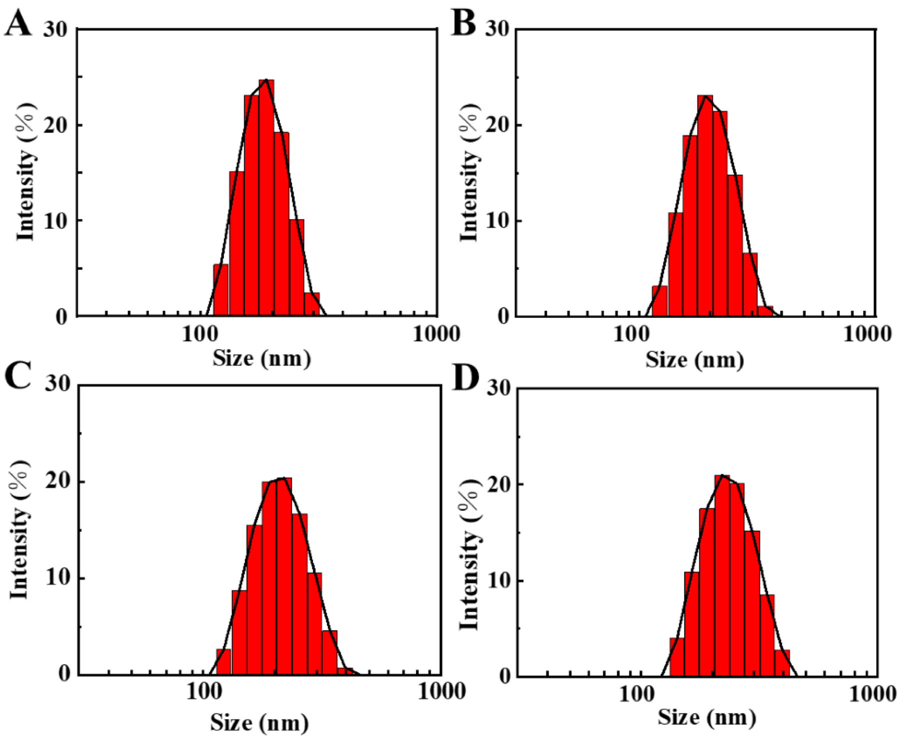

Supplementary Figure 2. (A), **(B)**, **(C)** and **(D)** show the particle size distributions of PB, PB/PM, PB/PM/HRP and PB/PM/HRP/Apt, respectively.

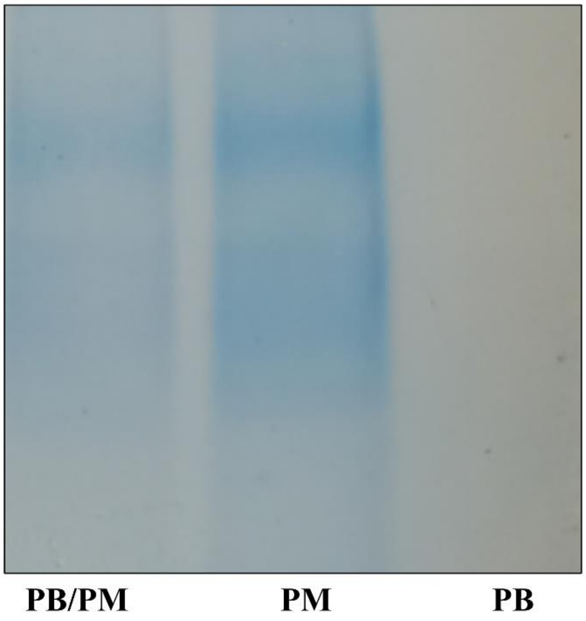

Supplementary Figure 3. SDS-PAGE images of PB, PM, and PB/PM.

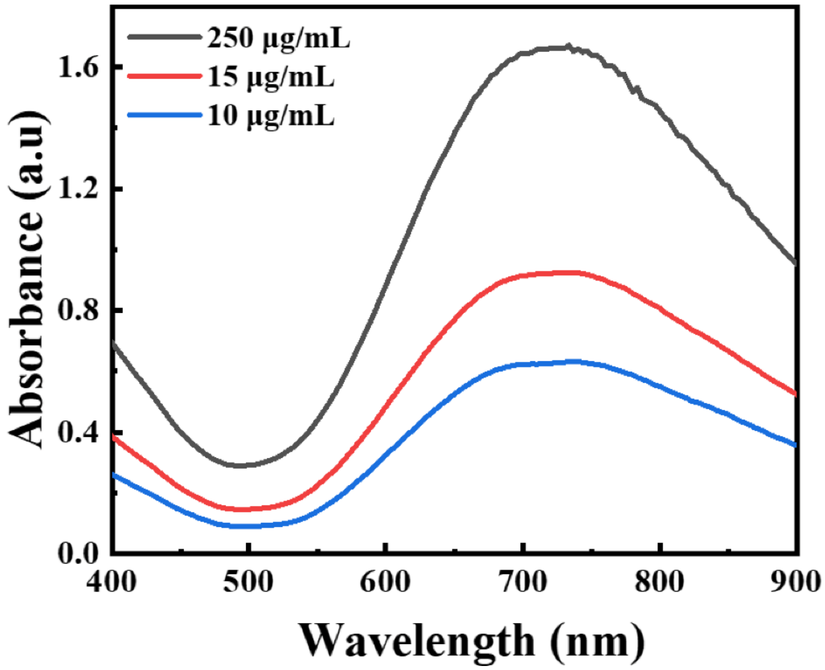

Supplementary Figure 4. UV-Vis absorption spectra of PB at different concentrations.

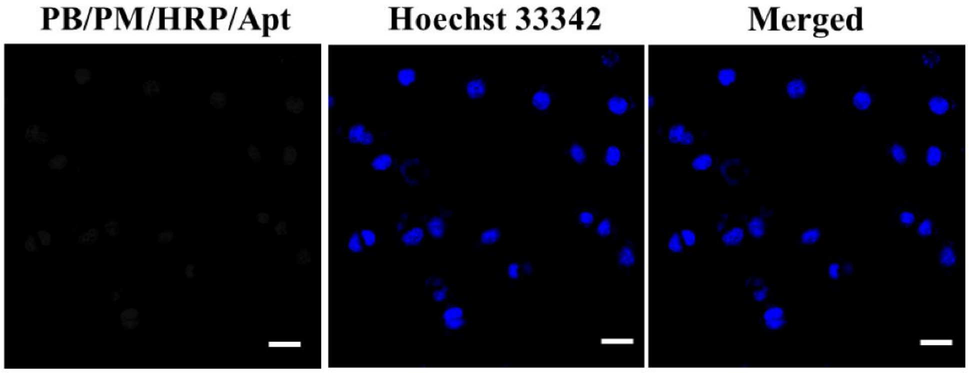

Supplementary Figure 5. Cellular internalization of PB/PM/HRP/Apt on MCF-7 cells.

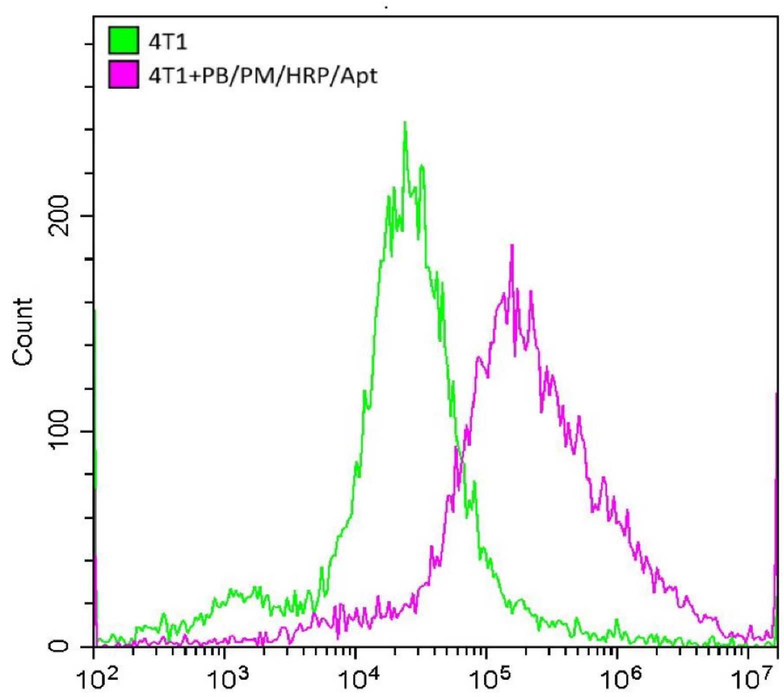

Supplementary Figure 6. Fluorescence detection of PB/PM/HRP/Apt in 4T1 cells.

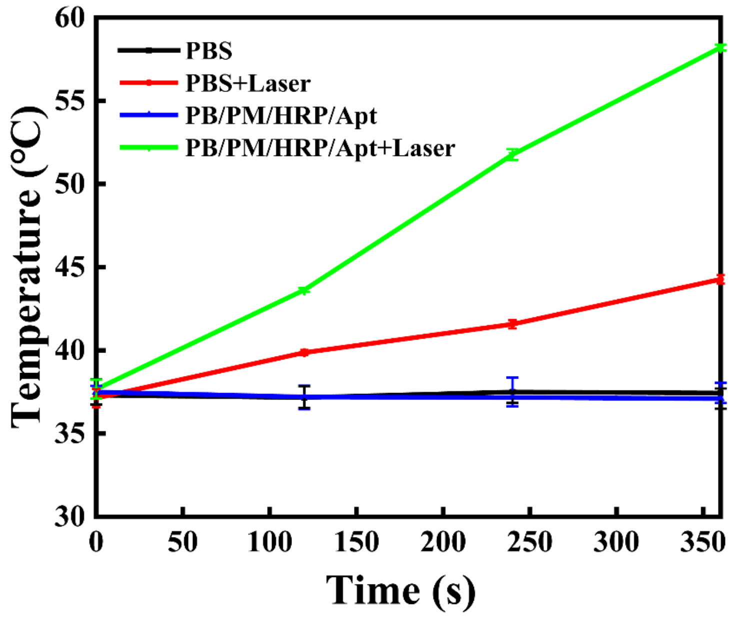

Supplementary Figure 7. Temperature rise curves of tumor-bearing mice (injection of PBS, injection of PBS+Laser, injection of PB/PM/HRP/Apt, injection of PB/PM/HRP/Apt+Laser).

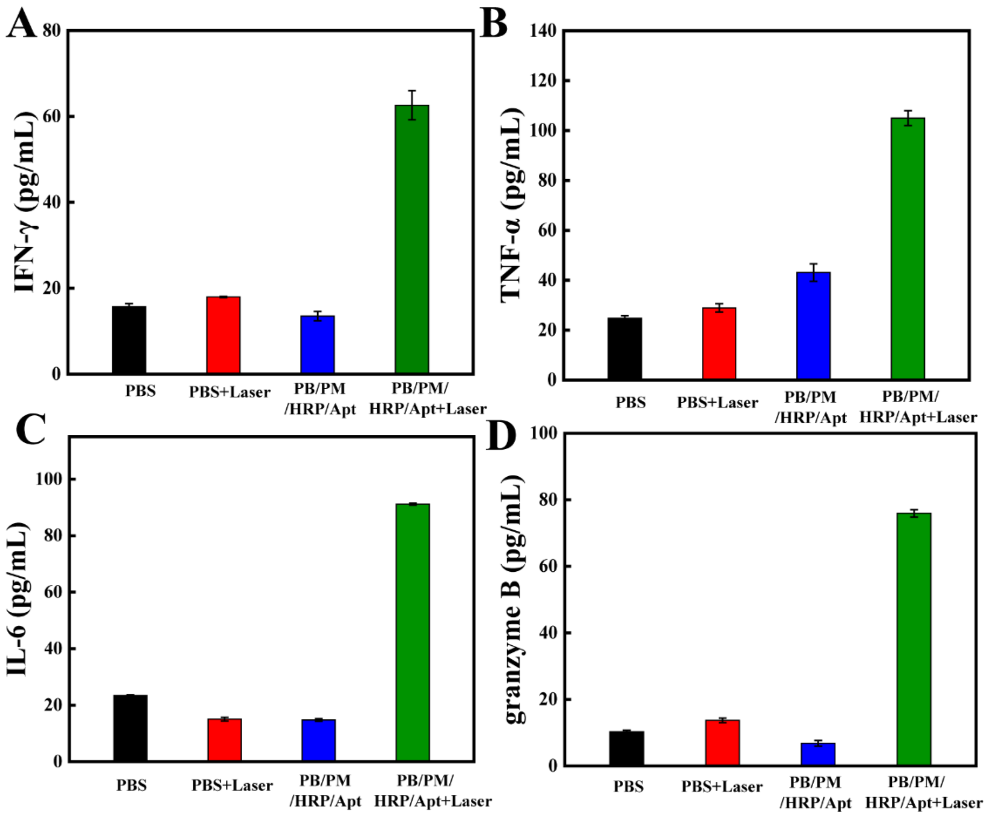

Supplementary Figure 8. ELISA results reflected the relative content changes of (A) IFN-γ, (B) TNF-α, (C) IL-6 and (D) granzyme B in tumor tissues.

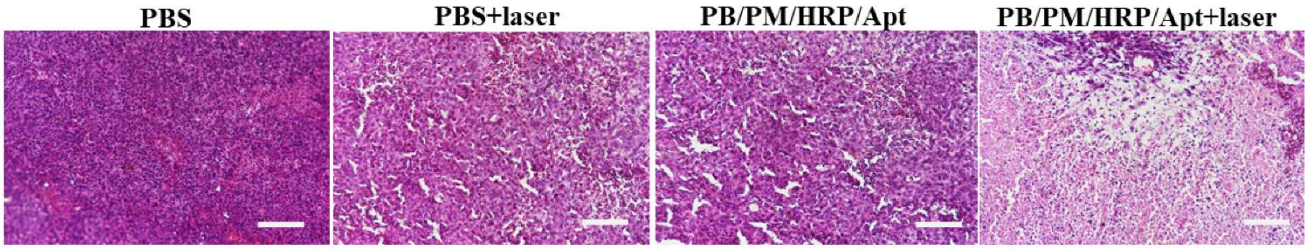

Supplementary Figure 9. H&E staining images of 4T1 solid tumor sections obtained after injection of PBS, PBS + Laser, PB/PM/HRP/Apt and PB/PM/HRP/Apt + Laser. Scale bar: 100 μm.
